# *In-vitro* effect of a single application of CPP-ACP pastes and different fluoridated solutions on the prevention of dental caries around orthodontic brackets

**DOI:** 10.1590/2177-6709.28.6.e2321383.oar

**Published:** 2024-01-08

**Authors:** Karla Lorene de França LEITE, Mariana Leonel MARTINS, Amanda Souza Nunes MONTEIRO, Thiago Isidro VIEIRA, Adílis Kalina ALEXANDRIA, Gustavo Miranda ROCHA, Andréa FONSECA-GONÇALVES, Matheus Melo PITHON, Yuri Wanderley CAVALCANTI, Lucianne Cople MAIA

**Affiliations:** 1 Universidade Federal do Rio de Janeiro, Faculdade de Odontologia, Departamento de Odontopediatria e Ortodontia (Rio de Janeiro/RJ, Brazil).; 2 Universidade Federal do Rio de Janeiro, Instituto de Biofísica Carlos Chagas Filho (Rio de Janeiro/RJ, Brazil).; 3 Universidade Estadual do Sudoeste da Bahia, Departamento de Saúde (Jequié/BA, Brazil).; 4 Universidade Federal da Paraíba, Departamento de Odontologia Clínica e Social (João Pessoa/PB, Brazil).

**Keywords:** Dentifrices, Fluorides, Orthodontic brackets, Dental biofilm

## Abstract

**Objective:**

To assess the *in-vitro* effect of single applications of CPP-ACP pastes and different fluoridated solutions on the prevention of dental caries around orthodontic brackets.

**Material and Methods:**

Tooth/bracket sets (n=65) were immersed in artificial saliva (1h at 37ºC) and randomly subjected to single applications (100µL; 1min) of casein phosphopeptide-amorphous calcium phosphate (CPP-ACP emulsion), CPP-ACP with fluoride (CPP-ACPF emulsion), solutions of titanium tetrafluoride (TiF_4_) or sodium fluoride (NaF), or no treatment (CG). Multispecies biofilm (5 x 10^5^ CFU/mL) was formed in the presence of 2% sucrose. After 24 h, the pH and the concentration of total soluble fluoride (TSF) were analyzed by culture medium. The presence of active white spot lesions (WSL) evaluated by macroscopic examination and the percent surface mineral loss (%SML) were analyzed. Also, the topography of enamel was detected by analysis of scanning electron microscopy (SEM). The data was assessed by chi-square, Kruskal-Wallis, and Mann-Whitney tests (p < 0.05).

**Results:**

Fluoride-containing compounds led to a smaller pH reduction than did CPP-ACP and CG (p<0.05). There was difference in TSF between the groups (p<0.05), denoted as TiF_4_> NaF > CPP-ACPF > CPP-ACP > CG. Regarding the presence of WSL and %SML, the NaF group obtained lower values (p<0.05), while TiF_4_ and CPP-ACPF were similar (p>0.05). SEM demonstrated that fluoride-free groups had a larger surface dissolution.

**Conclusion:**

Fluoridated groups including solutions and CPP-ACPF were more effective than CPP-ACP in reducing enamel demineralization around orthodontic brackets after a single application.

## RESUMO

**Objetivo:**

Avaliar *in-vitro* o efeito de uma aplicação única de cremes dentais de CPP-ACP e diferentes soluções fluoretadas na prevenção da cárie dentária ao redor de braquetes ortodônticos

**Material e Métodos:**

O conjunto dentes/braquetes (n=65) foi imerso em saliva artificial (1h em 37°C) randomizado e submetido a tratamento único (100µL; 1 min) de emulsão de fosfopeptídeo de caseína-fosfato de cálcio amorfo (CPP-ACP) e CPP-ACP associado ao flúor (CPP-ACPF); soluções de tetrafluoreto de titânio (TiF_4_) e fluoreto de sódio (NaF); e ausência de tratamento (GC). Biofilmes multiespécie (5 x 10^5^ CFU/mL) foram formados na presença de sacarose a 2%. Após 24h, o pH e a concentração de fluoreto solúvel total (FST) foram analisados pelo meio de cultura. Foram avaliadas a presença de lesões de mancha branca (LMB), por meio da análise de macroscopia visual, e a porcentagem de perda de dureza (%PD). Também foi verificada a topografia do esmalte, usando microscopia eletrônica de varredura (MEV). Os dados foram analisados pelos testes Qui-quadrado, Kruskal-Wallis e Mann-Whitney (p < 0,05).

**Resultados:**

Os compostos contendo flúor levaram a uma redução do pH menor do que o CPP-ACP e GC (*p*<0,05). Houve diferença no FST entre os grupos (*p* <0,05), sendo TiF_4_> NaF > CPP-ACPF > CPP-ACP > GC. Quanto à presença de LMB e à %PD, o grupo NaF obteve os menores valores (*p*<0,05), enquanto TiF_4_ e CPP-ACPF foram semelhantes (*p*> 0,05). A MEV demonstrou que os grupos sem flúor tiveram uma dissolução superficial maior.

**Conclusão:**

Os grupos fluoretados, incluindo soluções e CPP-ACPF, foram mais eficazes do que o CPP-ACP sem flúor na redução da desmineralização do esmalte ao redor dos braquetes ortodônticos após uma única aplicação.

## INTRODUCTION

Dental caries is a dysbiosis caused by the exposure of biofilm to fermentable carbohydrates.^1^ ^,^ ^2^ The pH of the medium reduces due to the release of acids by biofilm bacteria,^3^ leading to demineralization of the tooth structure. Clinically, white spot lesions (WSLs) develop on the tooth surface, which can turn into cavitated lesions if preventive or therapeutic measures are not taken.^4^

Patients using fixed orthodontic appliances are more prone to develop enamel carious lesions.^5^ These lesions occur due to enamel mineral loss caused by biofilm retention during orthodontic treatment, which is worsened by poor oral hygiene and high sugar intake.^6^ Orthodontically induced WSLs are one of the most common adverse effects of fixed orthodontic treatments, and dentifrices and fluoridated solutions are considered to control their evolution. However, several systematic reviews and meta-analyses assessing the effectiveness of such remineralization agents in controlling WSLs^5^ ^,^ ^7^ ^,^ ^8^ have concluded that there is a lack of consensus regarding the utility of these agents.

*In-vitro* studies have demonstrated that casein phosphopeptide-amorphous calcium phosphate (CPP-ACP) and titanium tetrafluoride (TiF_4_) are more effective against tooth demineralization than sodium fluoride (NaF) formulations.^9^ ^-^ ^12^ Further investigations are necessary to confirm these findings and disseminate evidence on newly developed products, which together may support future clinical trials. To gather evidence of the protective effects of CPP-ACP and TiF_4_ against cariogenic activity and biofilm retention, which would reduce the risk of caries in orthodontic patients, the present study aimed to the efficacy of a single application of CPP-ACP pastes and different fluoridated solutions on enamel surfaces around orthodontic brackets *in vitro*. Understanding the efficacy of different agents in controlling WSLs will promote the prevention of demineralization and facilitate remineralization. The null hypothesis for this study was that a single application of phosphopeptide-amorphous calcium phosphate (CPP-ACP), CPP-ACP with fluoride (CPP-ACPF), NaF or TiF_4_ on the enamel surfaces around orthodontic brackets would have a preventive effect on WSLs, with no difference between the agents used.

## MATERIAL AND METHODS

### STUDY DESIGN

This was an *in-vitro* study, in which the sample size calculation was based on the mean difference of tooth structure loss (TSL) in the TiF_4_ varnish group (30.52 ± 9.93) compared to negative control (52.08 ± 21.10 placebo varnish) observed in a previous study.^9^ Considering a power = 0.8, α = 0.05, and based on a two-sided test, a sample size of 11 blocks allocated into each group of treatment was required to complete the study. With 10% added to compensate for possible losses, at least 13 blocks for each group should be selected (G*Power version 3.1.9.2, Germany). This study considered a single application of experimental products: MI Paste^®^, MI Paste Plus^®^, and TiF_4_ and NaF solutions. Were considered independent data the outcomes as evaluations, pH analysis, measurement of total soluble fluoride (TSF), presence of white spot lesion (WSL) and enamel topography by scanning electron microscopy (SEM).

Dilution at a 1:3 (g/v) ratio using distilled and deionized water (Milli-Q Merck^®^, Saint Louis, USA)^13^ was performed to obtain the CPP-ACP emulsion (2% of CPP-ACP, 0 ppm of F^-^) and CPP-ACPF emulsion (2% CPP-ACPF, 900 ppm of F^-^) (MI Paste^®^ and MI Paste Plus^®^, CG America^®^, Illinois, USA). Fluoridated solutions were prepared with TiF_4_ (1% of TiF_4_, 6,135 ppm of F^-^) and NaF (1.36% of NaF, 6,135 ppm of F^-^) (Aldrich Chemical Co^®^, Saint Louis, USA). The fluoride concentration on these solutions groups were primarily defined according to the TiF_4_ group, since this substance is more difficult to synthetize. Therefore, the other solution was manipulated in order to present similar fluoride concentration to that observed in 1% TiF_4_ usually used in clinical practice. The groups were described as CPP-ACP, CPP-ACPF, TiF_4_, NaF and control group (CG), consisted of bacterial suspension.

### SPECIMEN PREPARATION

Bovine incisors without enamel defects were selected, and enamel blocks (8 x 8 x 2.5 mm) were obtained. The enamel blocks were cut, planed, and polished as proposed by Alexandria et al.^9^ Thereafter, a window measuring 19.63 mm², whose surrounding area was protected with a layer of acid-resistant varnish (Risqué^®^, São Paulo, Brazil) was exposed. To select the enamel blocks, the surface microhardness (SMH) of the dental enamel was assessed (in kgf/mm^2^) by a Knoop diamond indenter using a load of 50g/5s, with three equidistant indentations (100 µm) in the region close to the margin of the demarcated area. The selected blocks were within the 10% range in relation to the overall mean for initial microhardness. 

A single trained operator used the Transbond Plus Self Etching Primer (3M Unitek^®^, Monrovia, USA) and Transbond XT (3M ESPE^®^, Saint Paul, USA) for bonding the orthodontic bracket (3.20 x 2.70 mm) (Morelli, Sorocaba, Brazil) to the central area of the window. The orthodontic elastic band was used around the bracket to simulate the clinical situation.

### CARIOGENIC CHALLENGE

After random distribution (Microsoft Excel^®^), each treatment group included 13 tooth/bracket sets, which were placed in a 12-well polystyrene culture dish (model K12-024, Kasvi^®^, São José do Pinhais, Brazil), followed by sterilization under ultraviolet light (40 W) (t = 1 h).^14^

*Streptococcus mutans* (ATCC 25175), *S. salivarius* (ATCC 7073), *S. sanguinis* (ATCC 20556), and *Lactobacillus casei* (ATCC 393) strains, grown in TSB (Tryptic Soy Broth^®^; Oxoid, Hampshire, GBR) supplemented with 20% glycerol, were reactivated in Petri dishes (Alamar^®^, Diadema, Brazil) containing BHI (Brain Heart Infusion) agar (Difco^TM^, Sparks, Maryland, USA) and incubated in an oven at 37ºC for 48 h under microaerophilic conditions (5% of CO_2_). After that, the isolated bacterial colonies were suspended in BHI broth, and 4 to 6 h were allowed for their growth. 

The inoculum was standardized in compliance with the CLSI^15^ guidelines, and transferred to BHI broth containing 2% sucrose (pH=7.10) after homogenization, obtaining a final concentration of 5x10^5^ CFU/mL.

The cariogenic challenge was promoted by single application of test products around orthodontic brackets, in which one blinded researcher applied the test products only once (100 μL) in the intervention area around the orthodontic brackets (11 mm^2^) using a microbrush (KG Sorensen^®^, Cotia, Brazil) actively for 1 min in each enamel block. Afterwards, the specimens were subjected to acquired salivary pellicle formation following the model described by Amaechi et al.^16^ After removing the saliva, 5 mL of mixed inoculum (5 x 10^5^ CFU/mL) was added, followed by incubation at 37°C for 24 h. The culture medium was collected for pH analysis and measurement of TSF. The tooth/bracket sets were sonicated for 1 min and, subsequently, the brackets were debonded.

The control group, which did not undergo any treatment, consisted of bacterial suspension (multispecies biofilm of *Streptococcus spp.* and *Lactobacillus casei*) prepared in BHI broth containing 2% sucrose, for confirmation of viability of the strains and of the model used.

### ASSESSMENTS

The pH was measured in duplicate, using a microelectrode (PHOX^®^, Colombo, Brazil) calibrated with pH buffers of 4.0, 7.0, and 10.0.

TSF was quantified in all samples using a fluoride selective electrode coupled to a potentiometer (Orion Star Series, Thermo Fisher Scientific^®^, Waltham, USA) as proposed by Fernandez et al.^17^ TSF concentrations were measured with the supernatant of the culture medium, in which the samples had been stored for 24 h. The samples were read at the 1:1 (v/v) ratio of TISAB II.

The enamel surface was cleaned and dried, and evaluation of the WSL presence was performed by two calibrated researchers (ICC = 96%), who analyzed all the enamel surface samples, using opacity and roughness to determine the presence or absence of WSL around orthodontic brackets, by applying scores 0 (absence of WSL) and 1 (presence of WSL).^18^ A third researcher solved any disagreement between the two researchers.

Enlarged images of the exposed area were obtained with a stereomicroscopic (model 1005t, Opticam, São Paulo, Brazil) coupled to a digital camera (CMOS 10 megapixels, Opticam, São Paulo, Brazil), keeping the same scale for color, brightness, and light exposure.^19^ The presence, location, and different patterns of WSL were assessed in the different groups.

All specimens of each group were reassessed after cariogenic challenge by the same blinded and trained examiner to determine the final surface microhardness, in order to obtain the percentage of surface mineral loss (%SML) after the experiment.^20^ The %SML was calculated using the following equation:

%SML=(soundSMH–SMHafterin−vitrotreatment)/soundSMH*100.

Two specimens from each group were assessed by scanning electron microscopy (JEOL-JSM^®^; 6460LV, Tokyo, Japan). The area between the resin remnant after bracket debonding and the healthy enamel surface was examined. Photomicrographs were obtained at 5.000× and 20.000× magnification.

### STATISTICAL ANALYSIS

The data was analyzed using SPSS version 20.0 (IBM^®^, Chicago, USA) and the significance level was set at 5%. Normality was evaluated in all tested variables using the Shapiro-Wilk test. The Kruskal-Wallis and Mann-Whitney tests were used for pH, TSF and SML analyses. The Chi-Square and Fisher’s Exact test was used to assess the prevention of WSL by the tested product. 

## RESULTS

Fluoridated compounds caused a smaller pH reduction than those without fluoride and the control (p<0.05), but the solutions were more effective than CPP-ACP pastes (p<0.05). Regarding TSF, all groups differed between themselves (p<0.05), and TSF concentration in the TiF_4_ group was higher than in the NaF, CPP-ACPF, CPP-ACP, and control groups (Table 1). However, low test power (< 80%) was identified in the comparison analyzes between CPP-ACP and CG regarding pH. Therefore, it cannot be affirmed that CPP-ACP and CG were similar in terms of pH. For TSF, there was a statistically significant difference between the CPP-ACP and GC groups, with test power equal to 94.6%.

**Table 1:** Kruskal-Wallis and Mann-Whitney test results and distribution of quantitative variables as per pH and total soluble fluoride (TSF) concentrations [μg F^-^ / mL] for the experimental groups.

Groups	pH				TSF			
	Med	Min	Max	SD	Med	Min	Max	SD
CPP-ACP	4.12^A*^	4.06	4.27	0.06	0.17^A^	0.13	0.21	0.02
CPP-ACPF	4.26^C^	4.14	4.50	0.08	1.04^B^	0.65	1.31	0.1
TiF_4_	4.59^B^	4.47	4.88	0.1	20.5^C^	4.04	55.0	13.8
NaF	4.55^B^	4.46	4.73	0.08	10.9^D^	4.89	21.9	4.3
CG	4.12^A*^	4.05	4.17	0.03	0.14^E^	0.12	0.19	0.02

Med = median; Min = minimum; Max = maximum; SD = standard deviation. Different letters indicate statistical difference (p<0.05). *Although no statistical difference was identified, the test was low power (<80%).

**Figure 1:**
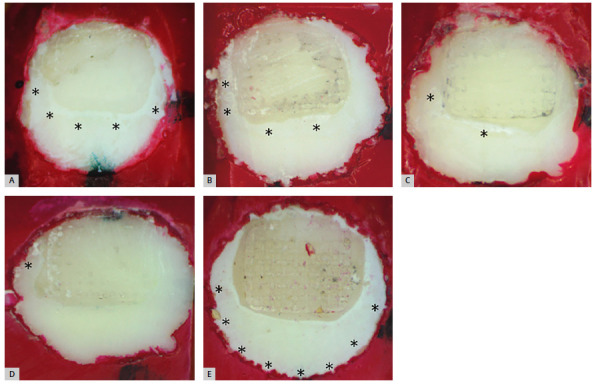
Representative images of active white spots in the exposed area of enamel blocks subjected to cariogenic challenge. The asterisk (*) indicates areas with WSL. A) CPP-ACP; B) CPP-ACPF; C) TiF_4_; D) NaF; and E) CG.

All specimens of the control group showed presence of WSL, whereas the NaF group showed the lowest rate. Only solutions differed from the control group (p<0.05), as both CPP-ACP and CPP-ACPF showed similar results to the control group in this regard (Tables 2 and 3). Different WSL patterns were observed in the experimental groups (Fig 1).

**Table 2:** Frequency of WSL in the experimental groups, by Fisher’s exact test.

Group	White Spot Lesion	
	Absence	Presence
CCP-ACP	7.7 % (n=1)	92.3 % (n=12)
CCP-ACPF	23.1 % (n=3)	76.9 % (n=10)
NaF	84.6 % (n=11)	15.3 % (n=2)
TiF_4_	53.8 % (n=7)	46.2 % (n=6)
CG	0.0 % (n=0)	100.0 % (n=13)

**Table 3:** Fisher’s exact tests with p-value of the difference, in presence/absence of white spot lesion in treatments.

Group	CG	CPP-ACP	CPP-ACPF	NaF	TiF_4_
CG	-	0.48	0.22	0.0001*	0.0052*
CPP-ACP	0.48	-	1.00	0.0048*	0.0968
CPP-ACPF	0.22	1.00	-	0.00169*	0.2262
NaF	0.0001*	0.0048*	0.00169*	-	0.411
TiF_4_	0.0052*	0.09680	0.2262	0.411	-

*Significant difference.

The fluoridated compounds had the best results, being that the NaF group obtained the lowest % SML (p < 0.05) than TiF_4_ and CPP-ACPF, which were similar (p > 0.05) and different from CPP-ACP (p < 0.05) (Table 4).

**Table 4:** Surface microhardness (SMH) analysis before and after the experiments and percentage of enamel surface microhardness loss.

Groups	SMH Before	SMH After	%SML
CPP-ACP	329,68^Aa^ ± 18,99	193,42^Ba^ ± 14,60	41,22^A^ ± 6,68
CPP-ACPF	335,73^Aa^ ± 9,47	212,75^Bb^ ± 31,59	36,52^B^ ± 9,97
TiF4	328,23^Aa^ ± 13,51	235,77^Bb^ ± 23,21	31,41^B^ ± 7,77
NaF	337,91^Aa^ ± 17,07	231,29^Bb^ ± 24,15	28,01^C^ ± 8,00
CG	332,77^Aa^ ± 18,16	117,21^Bc^ ± 27,19	64,49^D^ ± 9,09

Means followed by different letters are statistically different (p < 0.05). Uppercase letters show differences before and after the experiment in each group (paired samples *t* test, p < 0.05) and lowercase letters in the same column show differences between the treatments (Kruskal-Wallis and Mann-Whitney; p < 0.05).

**Figure 2:**
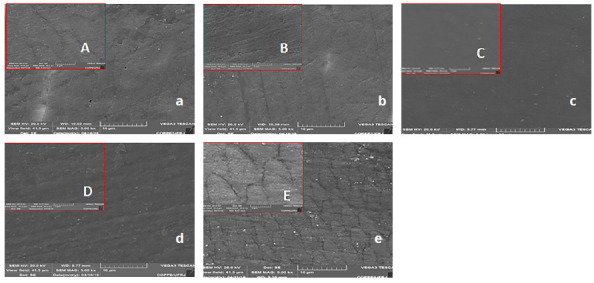
Surface of enamel blocks after treatment shown by scanning electron microscopy. Lowercase letters indicate 5,000x magnification and uppercase letters indicate 20,000x magnification. A) CPP-ACP; B) CPP-ACPF; C) TiF_4_; D) NaF; and E) CG.

The enamel surface topography differed between the groups, with decrease enamel dissolution in the TiF_4_, NaF, and CPP-ACPF groups, whereas larger exposure of enamel prisms was noted in the CPP-ACP and control groups (Fig 2).

## DISCUSSION

The present study aimed to assess the preventive effect of CPP-ACP pastes with or without fluoride and fluoridated solutions on dental caries on enamel surfaces around orthodontic brackets. Fluoridated solutions demonstrated preventive potential against incipient carious lesions, reducing the demineralizing potential of multispecies biofilms. This effect was also observed after the application of CPP-ACP containing fluoride, albeit to a lesser extent. Therefore, the null hypothesis was rejected since the tested products showed differences in their effect patterns.

This study attempted to overcome the limitations of an *in-vitro* assay by simulating biofilm retention using artificial saliva and orthodontic brackets. In addition, visual inspection of the tooth surface, which is a sensitive method for the diagnosis of carious lesions,^21^ was performed. This study did not consider the effect of toothbrushing; however, even without mechanical removal of the biofilm, the tested products proved to be effective in controlling demineralization. This is a limitation of the study, however, other studies have also reported methods similar to this one.^22^ ^,^ ^23^ It is important to highlight that the microbiological model used in the present study could indicate changes in pH values and the formation of WSLs within 24 h, as suggested in previous studies.^24^ ^,^ ^25^ Moreover, the single application of fluoridated products managed to delay demineralization, compared to the control group (no fluoride), as well as CPP-ACPF. In addition, even though bovine enamel is considered the substratum of choice for simulating human dental tissues in oral biofilm research^26^, studies using human teeth could provide more real-life simulations.

The use of preventive products may help managing patients im use of fixed orthodontic appliances, given that the major adverse effect of orthodontic treatment is the development of WSLs.^27^ ^,^ ^28^ In this regard, the present study showed that a single application of fluoridated and/or calcium-based products (CPP-ACP and CPP-ACPF) reduces the development of WSLs around brackets, making them good options for reducing enamel demineralization in the presence of orthodontic appliances. Thus, the use of these products should be promoted in the orthodontic routine.

In the present study, all groups significantly reduced the formation of WSL, compared with the control group, thereby indicating the ability of these products to minimize enamel demineralization. Nevertheless, fluoridated solutions outperformed CPP-ACP pastes. These findings probably have to do with the reactivity of the solutions, when compared to emulsions. Furthermore, the original fluoride concentration in the solutions (6135 ppm F) was higher than that in CPP-ACPF (900 ppm F). Even though the fluoride concentration differs between CPP-ACPF and solutions, this limitation may be overcome by the presence of calcium and phosphate ions in CPP-ACP dentifrice emulsions, which can interfere with enamel demineralization and remineralization processes,^29^ as observed, albeit to a lesser extent, in the present study. 

Oral hygiene instructions may be adapted according to the type of product used. In previous studies,^30^ ^,^ ^31^ CPP-ACP proved to be effective for enamel remineralization because of the synergistic effects of fluoride and calcium. Hence, everyday formulations that combine calcium and fluoride, either in CPP-ACP pastes or solutions, seem desirable not only for the prevention of dental caries around orthodontic brackets, but also for the remineralization of incipient lesions. Thus, future confirmatory studies on new products that combine both elements are needed.

The culture media in which enamel blocks were treated with NaF and TiF_4_ solutions had higher pH values and TSF concentrations than those in the other groups. This greater availability of fluoride in the medium might have reduced the formation and progression of WSLs. These findings could be attributed to the chemical characteristics of these compounds since NaF in healthy enamel produces fluoride reservoirs on the enamel surface, and TiF_4_, which forms an acid-resistant layer of titanium dioxide, provides additional protection against bacterial acids.^2^ ^,^ ^32^ ^,^ ^33^

As highlighted in previous studies,^24^ ^,^ ^25^ the presence of multispecies biofilm caused pH values to decrease to less than 4.5, favoring fluorapatite dissolution. In the present study, NaF and TiF_4_ treatments had a lower impact on pH in the culture medium, maintaining it above the level required for fluorapatite dissolution and thereby possibly contributing to less mineral loss in the enamel. Moreover, as shown by SEM, enamel prisms were more frequently observed in the control and CPP-ACP groups. Enamel integrity was maintained in formulations that contained NaF and TiF_4_, depending on their concentrations. These findings support the use of fluoridated solutions for the treatment of dental caries,^3^ especially those around orthodontic brackets.

## CONCLUSION

Fluoridated groups including solutions and CPP-ACPF were more effective than CPP-ACP in reducing enamel demineralization around orthodontic brackets after a single application. 

## References

[1] Simón-Soro A, Mira A (2015). Solving the etiology of dental caries. Trends Microbiol.

[2] Cury JA, Tenuta LMA (2009). Enamel remineralization controlling the caries disease or treating early caries lesions?. Braz Oral Res.

[3] Tenuta LMA, Cury JA (2010). Fluoride its role in dentistry. Braz Oral Res.

[4] Gorton J, Featherstone JD (2003). In vivo inhibition of demineralization around orthodontic brackets. Am J Orthod Dentofacial Orthop.

[5] Nascimento PL, Fernandes MT, Figueiredo FE, Faria-E-Silva AL (2016). Fluoride-Releasing Materials to Prevent White Spot Lesions around Orthodontic Brackets: A Systematic Review. Braz Dent J.

[6] Ren Y, Jongsma MA, Mei L, van der Mei HC, Busscher HJ (2014). Orthodontic treatment with fixed appliances and biofilm formation - a potential public health threat. Clin Oral Invest.

[7] Benson PE, Parkin N, Dyer F, Millett DT, Furness S, Germain P (2013). Fluorides for the prevention of early tooth decay (demineralised white lesions) during fixed brace treatment. Cochrane Database Syst Rev.

[8] Hu H, Feng C, Jiang Z, Wang L, Shrestha S, Yan J (2020). Effectiveness of remineralizing agents in the prevention and reversal of orthodontically induced white spot lesions a systematic review and network meta-analysis. Clin Oral Investig.

[9] Alexandria AK, Nassur C, Nóbrega CBC, Valença AMG, Rosalen PL, Maia LC (2017). In situ effect of titanium tetrafluoride varnish on enamel demineralization. Braz Oral Res.

[10] Oliveira PRA, Coutinho TCL, Portela MB, Paula VCA, Tostes MA (2017). Influence of biofilm formation on the mechanical properties of enamel after treatment with CPP-ACP crème. Braz Oral Res.

[11] Pithon MM, Dos Santos MJ, Andrade CS, Leão JC, Braz AK, de Araujo RE (2015). Effectiveness of varnish with CPP-ACP in prevention of caries lesions around orthodontic brackets an OCT evaluation. Eur J Orthod.

[12] Büyükyilmaz T, Tangugsorn V, Ogaard B, Arends J, Ruben J, Rølla G (1994). The effect of titanium tetrafluoride (TiF4) application around orthodontic brackets. Am J Orthod Dentofacial Orthop.

[13] De Souza CC, Cury JL, Coutinho TC, Da Silva EM, Tostes MA (2014). Effect of different application frequencies of CPP-ACP and fluoride dentifrice on demineralized enamel a laboratory study. Am J Dent.

[14] Katara G, Hemvani N, Chitnis S, Chitnis V, Chitnis DS (2008). Surface disinfection by exposure to germicidal UV light. Indian J Med Microbiol.

[15] CLSI (2012). Performance Standards for Antimicrobial Susceptibility Testing; Twenty-Second Informational Supplement. CLSI document M100-S22.

[16] Amaechi BT, Higham SM, Edgar WM (1999). Techniques for the production of dental eroded lesions in vitro. J Oral Rehabil.

[17] Fernández CE, Tenuta LMA, Paulo Zárate P, Cury JA (2014). Insoluble NaF in Duraphat(r) may prolong fluoride reactivity of varnish retained on dental surfaces. Braz Dent J.

[18] Passalini P, Fidalgo TK, Caldeira EM, Gleiser R, Nojima Mda C, Maia LC (2010). Preventive effect of fluoridated orthodontic resins subjected to high cariogenic challenges. Braz Dent J.

[19] Neves AA, Vargas DOA, Santos TMP, Lopes RT, Sousa FB (2016). Is the morphology and activity of the occlusal carious lesion related to the lesion progression stage. Arch Oral Biol.

[20] Cury JA, Rebelo MAB, Del Bel Cury AA, Derbyshire MTVC, Taubchoury CPM (2000). Biochemical composition and cariogenicity of dental plaque formed in the presence of sucrose of glucose and fructose. Caries Res.

[21] Biesbrock AR, Chesters RK, Ellwood RP, Smith SR (2004). The challenges of validating diagnostic methods relative to a conventional two-year caries clinical trial. J Dent Res.

[22] Fernández CE, Tenuta LM, Cury JA (2016). Validation of a cariogenic biofilm model to evaluate the effect of fluoride on enamel and root dentine demineralization. PLoS One.

[23] Ccahuana-Vásquez RA, Cury JA (2010). S. mutans biofilm model to evaluate antimicrobial substances and enamel demineralization. Braz Oral Res.

[24] Takahashi N, Nyvad B (2011). The role of bacteria in the caries process ecological perspectives. J Dent Res.

[25] Jiang S, Chen S, Zhang C, Zhao X, Huang X, Cai Z (2017). Effect of the biofilm age and starvation on acid tolerance of biofilm formed by Streptococcus mutans isolated from caries-active and caries-free adults. Int J Mol Sci.

[26] Teruel Jde D, Alcolea A, Hernández A, Ruiz AJ (2015). Comparison of chemical composition of enamel and dentine in human, bovine, porcine and ovine teeth. Arch Oral Biol.

[27] Beerens MW, Boekitwetan F, van der Veen MH, ten Cate JM (2015). White spot lesions after orthodontic treatment assessed by clinical photographs and by quantitative light-induced fluorescence imaging; a retrospective study. Acta Odontol Scand.

[28] van der Kaaij NC, van der Veen MH, van der Kaaij MA, ten Cate JM (2015). A prospective, randomized placebo-controlled clinical trial on the effects of a fluoride rinse on white spot lesion development and bleeding in orthodontic patients. Eur J Oral Sci.

[29] Hara AT, Karlinsey RL, Zero DT (2008). Dentine remineralization by simulated saliva formulations with different Ca and Pi contents. Caries Res.

[30] Ogata K, Warita S, Shimazu K, Kawakami T, Aoyagi K, Karibe H (2010). Combined effect of paste containing casein phosphopeptide-amorphous calcium phosphate and fluoride on enamel lesions: an in vitro pH-cycling study. Pediatr Dent.

[31] Cochrane NJ, Saranathan S, Cai F, Cross KJ, Reynolds EC (2008). Enamel subsurface lesion remineralisation with casein phosphopeptide stabilised solutions of calcium, phosphate and fluoride. Caries Res.

[32] Ribeiro CC, Gibson I, Barbosa MA (2006). The uptake of titanium ions by hydroxyapatite particles-structural changes and possible mechanisms. Biomaterials.

[33] Wei SH, Soboroff DM, Wefel JS (1976). Effects of titanium tetrafluoride on human enamel. J Dent Res.

